# Circulating ECVs prevent neurodegeneration and preserve neuronal function in a model of preclinical intracerebral hemorrhage

**DOI:** 10.1016/j.omtn.2023.04.022

**Published:** 2023-05-12

**Authors:** Annette Burkhart, Torben Moos

**Affiliations:** 1 Neurobiology Research and Drug Delivery, Department of Health Science and Technology, Aalborg University, Aalborg, Denmark

## Main text

The use of extracellular vesicles (ECVs) as therapeutics is coming of age with an increasing level of studies emerging demonstrating effects in a broad variety of preclinical models. In this issue, Laso-García et al.^1^ report on beneficial effects in the treatment of intracerebral hemorrhage (ICH) using an experimental model with ECVs injected into peripheral circulation.

**Figure 1:**
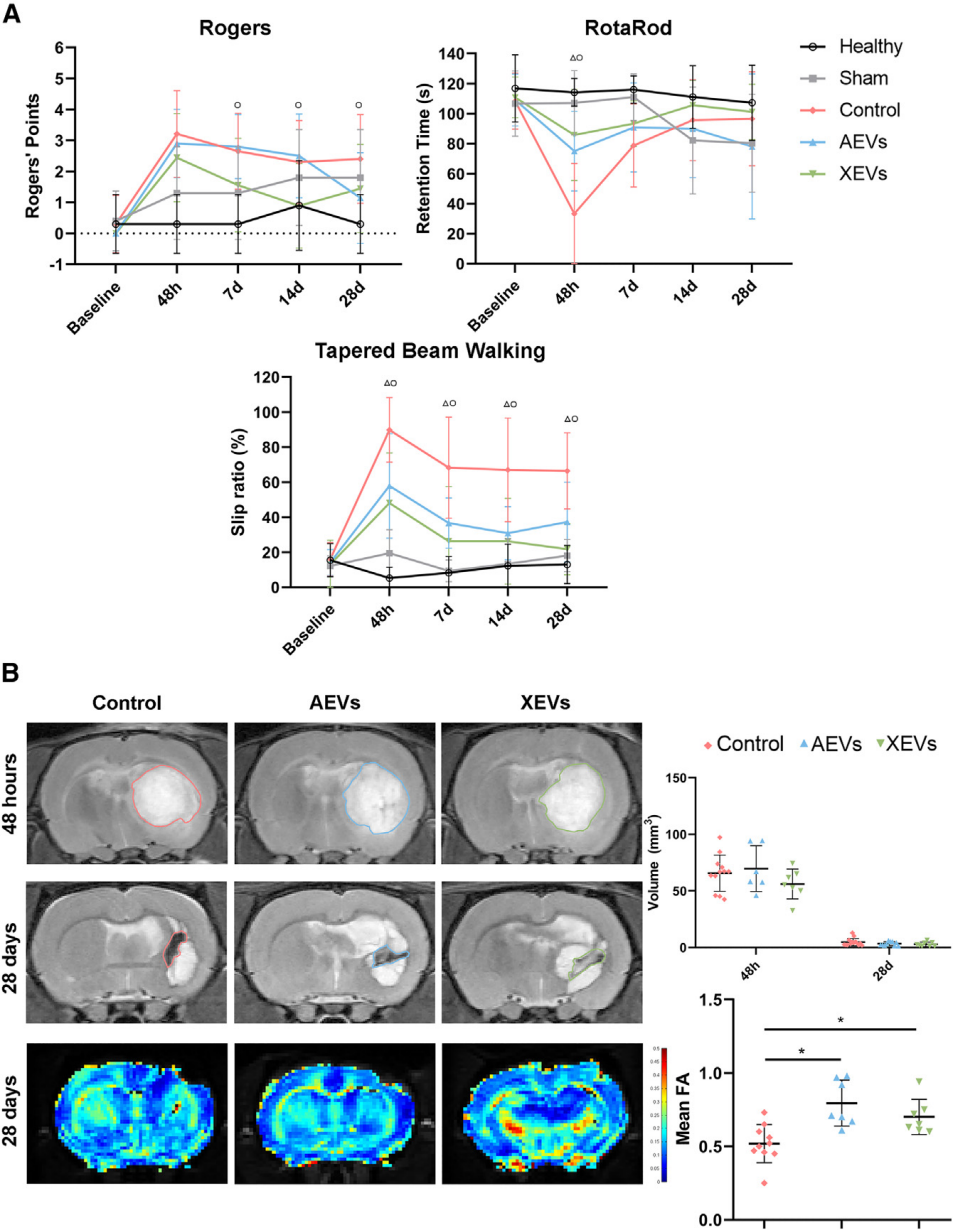
Functional evaluation and brain imaging after treatment with extracellular vesicles in experimental hemorrhagic stroke (A) Functional evaluation tests along the study time points. “Δ” indicates p *<* 0.05 in AEV-treated animals compared with the control group. “O” indicates p *<* 0.05 in the XEVs-treated animals compared with the control group. The motor recovery of the animals treated with AEVs and XEVs was significantly better than that of the control group, as shown in all tests used in the study. (B) T2-MRI images. Colored lines mark the total volume of lesion at 48 h and the residual lesion at 28 days surrounded by dilated cisterns because of tissue retraction. The DTI-FA values were significantly higher in the treated animals compared with the controls at 28 days, indicating greater white matter integrity after either AEV or XEV treatment. ∗p *<* 0.05. Data are shown as mean ± SD. Abbreviations are as follows: AEVs, allogenic extracellular vesicles; FA, fractional anisotropy; ICH, intracerebral hemorrhage; XEVs, xenogeneic extracellular vesicles. Legends adapted and slightly modified from Laso-García et al.^1^

ECVs were previously considered a disposal of unused or harmful RNA, proteins, and lipids but are now considered important as secreted vesicles acting as carriers of cargos for intercellular exchange.^2^^,^^3^ They are formed from bilayer lipid membranes. They are released by all living cells and consist of a variety of subtypes like exosomes, microvesicles, and apoptotic bodies.^2^^,^^3^ Using a preclinical model, the publication of Laso-García and co-workers^1^ now shows how intravenously injected ECVs aid in recovering from brain damage in an experimental model of ICH. To induce ICH, collagenase, which disrupts the integrity of the vascular basement membrane, was injected stereotactically into the rat striatum. This was followed by injection of isolated ECVs after 24 h using ECVs isolated from serum of either allogenic rats or humans with spontaneous recovery after ICH. The ECVs were well characterized by means of measures of protein content and morphology, including electron microscopic analysis, revealing sizes <200 nm. Injected in doses of approximately 100 μg total protein, the ECVs improved motor functions in rats in behavioral scores examined after 28 days (Figure 1). At the cellular level, ECVs studied for their content of proteins also after 28 days indicated preserved projecting fibers of the perilesional region, maintained myelination, and ameliorated gliosis. The analyses of the content of ECVs also revealed downregulation of markers of immune system activation pathways following injection into rats of xenogeneic ECVs from humans suggesting that the ECVs were able to revert post-stroke immunosuppression.

Laso-García and co-workers^1^ included two principal populations of ECVs for their study of therapeutic efficacy in the rats with experimentally induced ICH. One set included ECVs isolated from peripheral blood of normal rats, while the other involved ECVs isolated from peripheral blood of humans, who had overcome an ICH particularly well. Characteristically, in both experimental situations, the cellular origin of the ECVs is quite difficult to disclose, and this uncertainty is clearly of further relevance for pursuit, not at least to learn which cell types are responsible for the release of these beneficial ECVs reported here. The ECVs from patients who recently overcame an ICH were probably released from the brain as indicated by their content of neuronal proteins, but this remains to be further confirmed.

It has been known for some time that following pathological conditions in the brain, e.g., in ischemic stroke and traumatic brain injury, ECVs are released from the brain into peripheral circulation and probably also contribute to promote an acute-phase response and exaggerated inflammation both in brain and the periphery.^4^^,^^5^^,^^6^ In ischemic stroke, these ECVs derive from the endothelial cells, which enhance their secretion of ECVs. Unfortunately, such ECVs may have deteriorating effects on the remaining body and lead to co-morbidity.^4^^,^^5^^,^^6^ Interestingly, the ECVs used in the study of Laso-García and co-workers^1^ demonstrate opposite effects, which clearly warrants further studies of the content of ECVs in ICH relative to ECVs isolated from patients with a poorer outcome, not at least with a delineation of the activation of the immune system.

The study of Laso-García and co-workers^1^ implies a novel principle for therapy in ICH based on treatment with circulating ECVs. In spite of the study involving injection of ECVs of exogenous origin, the ECVs could principally also derive from self-induced synthesis in brain following ICH. Further knowledge is also needed to understand if brain releases ECVs and from which cell types in ICH, e.g., candidate cells for ECV release should be identified, the synthesis and release of ECVs from such cells should be characterized and compared in between normal and pathological conditions with respect to the of contents of RNA, proteins, and lipids.

In the context of understanding the significance of the ECVs, it is interesting that Laso-García and co-workers^1^ demonstrate that the content of these vesicles suggested neuroprotection of both gray and white matter regions. Following ICH, the vascularization of the brain area is temporarily compromised due to raised intracranial pressure.^7^ Later, edema formation may also reduce the vascular blood flow in the area with ICH, which limits the entry of ECVs. Mechanistically, the ECVs probably slip into the affected areas affected early in ICH before edema formation occurs. The ECVs then bypass the restraints of the blood-brain barrier, hence providing their accessibility for neurons, including their projection fibers, and glia cells like astrocytes and microglia, both important for the resulting gliosis in ICH.^8^^,^^9^

The study of Laso-García and co-workers^1^ has opened a new vista on the applicability of ECVs as a potential therapeutic for treatment of patients with hemorrhagic stroke. Future studies should focus on methods to unravel the release of ECVs from the brain and elsewhere, which cell types are responsible for the release of ECVs, whether the content of ECVs changes in cerebrovascular pathology, how they distribute in the body, and if their content discloses specific bioactive candidates relevant for specific molecular therapy. The study may also inspire further pursuit of cellular therapeutic approaches in ICH by using progenitor cells of the brain engineered to display a characteristic profile of secreted ECVs. This approach was recently taken showing an improved outcome in ischemic stroke.^10^^,^^11^
